# A low-voltage-driven MEMS ultrasonic phased-array transducer for fast 3D volumetric imaging

**DOI:** 10.1038/s41378-024-00755-9

**Published:** 2024-09-12

**Authors:** Yun Zhang, Tong Jin, Yining Deng, Zijie Zhao, Rui Wang, Qiong He, Jianwen Luo, Jiawei Li, Kang Du, Tao Wu, Chenfang Yan, Hao Zhang, Xinchao Lu, Chengjun Huang, Hang Gao

**Affiliations:** 1 Institute of Microelectronics of the Chinese Academy of Sciences, 100029 Beijing, China; 2 University of Chinese Academy of Sciences, 100049 Beijing, China; 3 School of Biomedical Engineering, Tsinghua University, 100084 Beijing, China; 4 School of Information Science and Technology, ShanghaiTech University, 201210 Shanghai, China

**Keywords:** Electrical and electronic engineering, Biosensors, Environmental, health and safety issues

## Abstract

Wearable ultrasound imaging technology has become an emerging modality for the continuous monitoring of deep-tissue physiology, providing crucial health and disease information. Fast volumetric imaging that can provide a full spatiotemporal view of intrinsic 3D targets is desirable for interpreting internal organ dynamics. However, existing 1D ultrasound transducer arrays provide 2D images, making it challenging to overcome the trade-off between the temporal resolution and volumetric coverage. In addition, the high driving voltage limits their implementation in wearable settings. With the use of microelectromechanical system (MEMS) technology, we report an ultrasonic phased-array transducer, i.e., a 2D piezoelectric micromachined ultrasound transducer (pMUT) array, which is driven by a low voltage and is chip-compatible for fast 3D volumetric imaging. By grouping multiple pMUT cells into one single drive channel/element, we propose an innovative cell–element–array design and operation of a pMUT array that can be used to quantitatively characterize the key coupling effects between each pMUT cell, allowing 3D imaging with 5-V actuation. The pMUT array demonstrates fast volumetric imaging covering a range of 40 mm × 40 mm × 70 mm in wire phantom and vascular phantom experiments, achieving a high temporal frame rate of 11 kHz. The proposed solution offers a full volumetric view of deep-tissue disorders in a fast manner, paving the way for long-term wearable imaging technology for various organs in deep tissues.

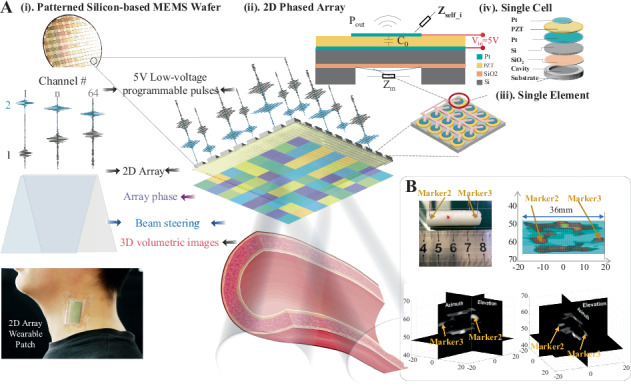

## Introduction

Wearable ultrasound devices that enable noninvasive, continuous imaging of deep tissues could significantly contribute to the early detection and surveillance of various diseases. Moreover, fast 3D volumetric imaging can facilitate full-volume acquisition within a short period, allowing improved accuracy and reproducibility over 2D methods for automated surface extraction and quantification, real-time transesophageal imaging, and accurate navigation within the 3D volume^1^. For example, the complex nature of the cardiovascular system can render thorough anatomic evaluation of malformations challenging. In such cases, the perspective given by fast volumetric imaging can provide a complete assessment of the extent and severity of valvular or other abnormalities during diagnosis. Additionally, continuous monitoring can aid in understanding such pathophysiological or other conditions across an extended timeline and thus enables timely and accurate medical interventions to achieve better outcomes. However, existing ultrasound methods for imaging deep tissue enable either continuous monitoring or fast volumetric acquisition, but not both^2,3^.

Conventional piezoelectric array transducers, which are suitable for volumetric imaging, exhibit limited applications in continuous monitoring owing to device bulkiness and fabrication^4^. Normally, conventional piezoelectric array transducers operate in the isotropic thickness extension mode, where the impedance mismatch between the transducer material and the acoustic medium at interfaces causes the reflection of acoustic waves, resulting in a significant loss of acoustic energy^5,6^. Hence, an acoustic impedance matching layer akin to human tissues has been added^7^. Recently, piezoelectric composites surrounded by epoxy/polymer filling material, despite enhanced electromechanical coupling coefficients, still require a drive voltage higher than 25 V to achieve a notable penetration depth^8,9^ or merely allow the acquisition of 1D signals at a low voltage^10^. Moreover, mechanical dicing in fabrication restricts pitch dimensions and causes the aliasing phenomenon^7^. As a result, the above coupling problems limit the array-filling ratio of high-performance components, precluding the development of wearable systems that require a high integration density at a low power consumption. Facilitated by microelectromechanical system (MEMS) technology, micromachined ultrasonic transducers (MUTs) have been developed with the advantages of miniaturized footprints and easy array fabrication compatible with drive-integrated circuits^4,11^, including capacitive micromachined ultrasound transducers (cMUTs)^12^ and piezoelectric micromachined ultrasound transducers (pMUTs)^12^. cMUTs typically exhibit large bandwidths and high electromechanical coupling coefficients, providing significant advantages in medical imaging and high-frequency applications^13^. However, these advantages are counterbalanced by several limitations. cMUTs require electrostatic actuation to maintain small gap heights, necessitating high DC bias voltages. Additionally, the transduction process of cMUTs is nonlinear^14^, potentially resulting in a reduced acoustic power output. This decrease in acoustic power can lead to performance degradation, particularly with increasing imaging depth^15^. Moreover, the application of high DC voltages poses safety concerns and exhibits significant challenges in circuit design. Compared to cMUTs^16–19^, pMUTs do not require a high bias voltage due to their passivity and thus are more suitable for wearable applications^14,20–22^. pMUTs operating in the flexural vibration mode offer a lower acoustic impedance, and thus, it is easier to achieve a high acoustic coupling efficiency for long-term wearable use^23^. Moreover, how to drive high-density pMUT arrays in an efficient way is receiving increasing attention. In an array layout, the interactions between pMUT cells across the electrical, mechanical, and acoustic domains are referred to as coupling effects, also denoted as crosstalk^24,25^. When operated for medical imaging, acoustic coupling dominates due to the damping vibration and increasing radiation pressure^26^.

To quantitatively characterize coupling effects, equivalent circuit (EQC) models have been developed for pMUT array design. The mutual radiation impedance and self-radiation impedance of each device are projected into the acoustic domain, accounting for acoustic coupling in the transmit and receive modes for pMUT arrays^26,27^. However, their applications in medical imaging are still at an early stage, facing the challenges of insufficient quantitative and experimental validation^28^. For instance, Akhbari et al.^26^ established equivalent circuit models for circular and curved PMUT single elements and arrays and successfully analyzed the output performance of the array. Xu et al.^29^ reported an equivalent circuit model for a resonant cavity-based PMUT array and optimized the design with the model to achieve a high output pressure. However, these works do not include simulations and preclude any validation studies with experiments toward medical imaging applications. As the size of high-density pMUT arrays increases, driving each pMUT cell through an individual channel remains difficult due to the complexity of the integrated circuit system. As such, grouping pMUT cells into one element for each drive would render the array scalable, dramatically reducing the number of drive elements and allowing ultrasound-on-chip design with low-power integrated circuits (i.e., complementary metal-oxide semiconductor, CMOS). Considerable research has focused on grouping pMUT cells into elements to achieve high-density arrays for excitation purposes. For instance, Zheng et al.^30^ arranged 2520 cells into single elements to construct square- and ring-shaped pMUT arrays for photoacoustic endoscopy applications. Dausch et al.^31^ reported a 256-channel pMUT array intended for intracardiac ultrasound imaging, where every four elements were connected to form a single channel. Moreover, Cheng et al.^32^ designed a pMUT array for particle manipulation, where each channel consists of 20 cells connected electrically in parallel. However, none of the aforementioned studies focused on developing a theoretical analytical model to assess the performance of arrays for group driving. Moreover, acoustic crosstalk, which occurs not only between cells but also between elements, complicates quantitative analysis. To the authors’ knowledge, full volumetric imaging results with low-voltage pMUT arrays have never been reported. For in-air use, the 104.5-kHz pMUT array proposed by Xia et al.^33^, operated at a voltage of 2 V for emitting ultrasound in air, falls beyond the scope of monitoring and imaging systems that necessitate both transmit and receive capabilities for signal acquisition. Due to the inherent requirement of cMUTs for a high DC bias voltage, the work of Goel and Merbeler^34,35^ focused primarily on reducing this DC bias voltage. Although the driving DC voltage can be reduced to as low as 7.4 V^35^, specific details regarding the AC voltage and a corresponding demonstration of in-air portable application were not provided. In contrast to air-coupled ultrasound transducers, medical array transducers exhibit challenges such as operating at higher frequencies within the megahertz range and effectively capturing weak scattered signals from the targeted tissues. As indicated in Table S1, the reviewed excitation voltages of the piezoelectric transducers used for tissue monitoring or imaging exceeded 20 V, including conventional bulk PZT transducers and 1–3 composite PZT transducers^2,8,10,36,37^, as well as pMUT arrays for intracardiac imaging^31^ and harmonic imaging or image-guided therapy^38^ (refer to Table S1 in the Supplementary Materials for details).

In summary, quantitative studies on scalable pMUT arrays for 3D imaging applications are lacking. In contrast to the existing state-of-the-art approaches, we introduce a novel hierarchical analytical equivalent circuit (EQC) model for 2D phased-array pMUT transducers. This model enables quantitative performance analysis of individual cells, particularly focusing on the crosstalk phenomenon between cells when they are connected in parallel to form a driving channel (element). Importantly, our model allows for numerical assessment of the coupling performance both within and between the elements as the 2D phased array is expanded, which is crucial for achieving low-voltage-driven 3D volumetric imaging.

**Fig. 1:**
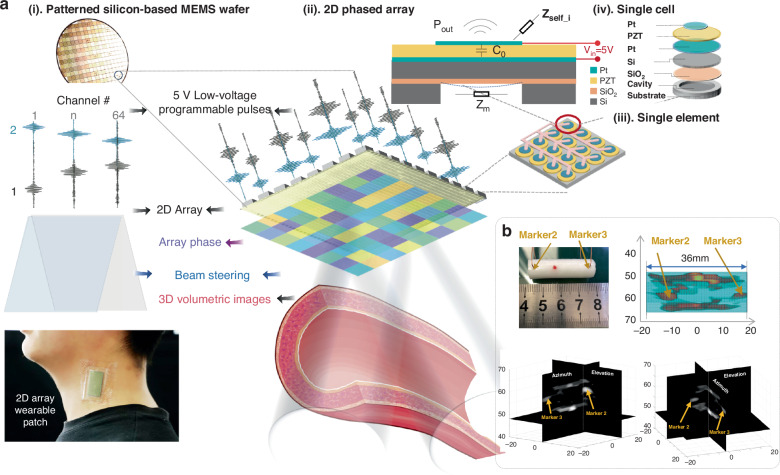
Schematics of the low-voltage-driven MEMS ultrasonic phased-array transducer and representative 3D volumetric imaging results for vascular phantoms. **a** Schematic of the 2D MEMS ultrasonic phased-array transducer enabling beamforming with programmable pulses in 64 elements and 3D volumetric ultrasound imaging of the cardiovascular system. The top view shows the proposed cell (**iv**)–element (**iii**)–array (**ii**) transducer design flow based on multilayer processing technology on a microelectromechanical system (MEMS) wafer **(i)**, which benefits the low-voltage drive and 2D array imaging patch in a wearable manner. **b** Detailed images of the 3D vascular phantom and 3D volumetric images covering 40 mm × 40 mm × 70 mm at a frame rate of 11 kHz acquired by the 2D MEMS ultrasonic phased-array transducer. The two orthogonal images derived from the 3D volumetric results show the profile of the vascular phantom in the azimuth and axial dimensions with wire markers 2 and 3 highlighted in yellow

In this paper, flexural-mode pMUT cells are grouped in parallel into one drive channel, i.e., an element, and a 2D array layout is designed as comprising 8 × 8 elements, allowing for programmable phases of 5-V pulses at each channel to facilitate 3D transmit/receive beamforming. This approach enhances the performance of low-voltage-driven pMUT transducers for fast volumetric imaging of deep tissue in a wearable manner. As shown in Fig. 1, we propose a novel EQC methodology, namely, the multilevel cell–element–array model, to quantitatively characterize the performance of pMUT phased arrays, especially the dominant acoustic coupling effects. Thereafter, we experimentally validated the cell–element–array model, including its electrical, mechanical and acoustic characteristics. The volumetric imaging performance is demonstrated in wire phantom and vascular phantom experiments and characterized in terms of the image penetration depth and temporal performance. In summary, in this study, the mechanism of the low-voltage pMUT phased-array ultrasonic transducer is reported, together with its design, fabrication, characterization, and full volumetric imaging results.

## Materials and methods

### Design, fabrication, and working principle of the MEMS ultrasonic phased-array transducer

**Fig. 2:**
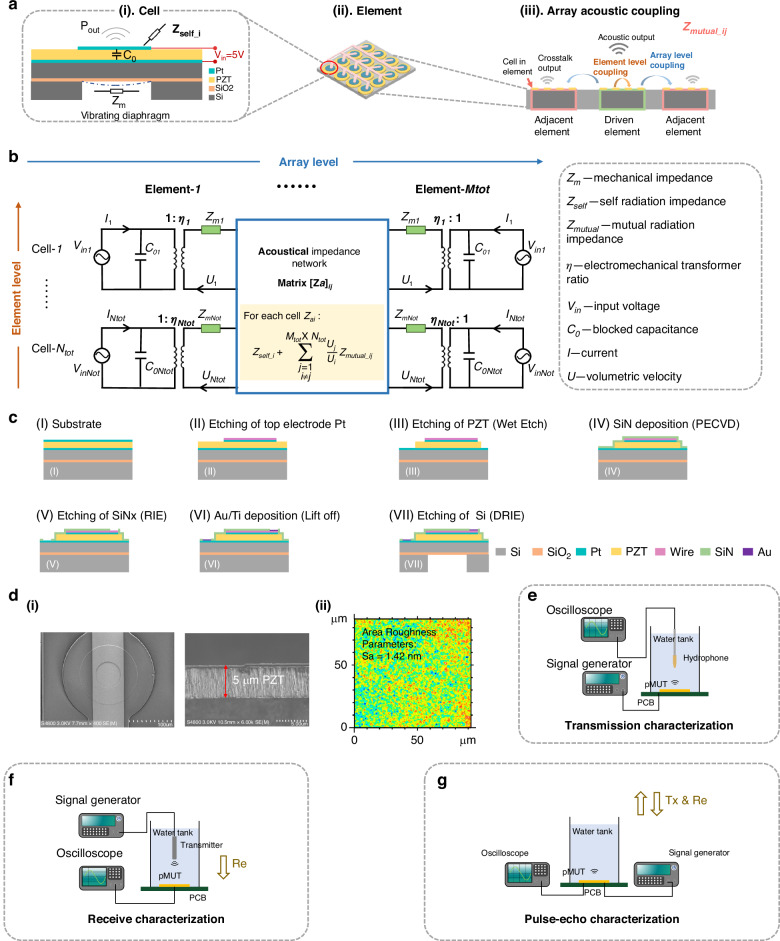
Schematic of the acoustic coupling within MEMS phased-array transducers and equivalent circuit (EQC) modeling of the electrical, mechanical and acoustic interactions at the element and array levels. **a** Detailed illustration of acoustic coupling in the cell–element–array design for the proposed MEMS phased-array transducer. (**i**) The top shows a cross-sectional view of multilayer pMUT cell *i* driven by 5-V voltage *V*_*in*_, characterized by the electrical capacitance *C*_*0*_, the mechanical impedance *Z*_*m*,_ the acoustic impedance *Z*_*self_i*_ and the transmit pressure *P*_*out*_. (**ii**) Each element consisting of a 4 × 4 cell is actuated by a single channel in the array. (**iii**) Array acoustic coupling effects *Z*_*mutual_ij*_, also referred to as crosstalk, include the acoustic interactions not only between the active cells within the driven element *i* (element-level coupling) but also between the driven element *i* and each adjacent element *j* (array-level coupling). **b** Detailed equivalent circuit model (EQC) representation of MEMS phased-array transducers, where *N*_*tot*_ cells are grouped as one element, and *M*_*tot*_ elements are driven by *M*_*tot*_ actuations as one array. For each pMUT cell, the energy flow current *I* in the electrical domain can change to reach the volumetric velocity of the vibrated membrane *U* in the mechanical domain, the efficiency of which is characterized as the electromechanical transduction ratio $\eta$. The acoustic impedance network ${Z}_{{ai}}$ of active cell *i* includes *Z*_*self_i*_ and *Z*_*mutual_ij*_, which denote the acoustic coupling effects contributed by all adjacent cells *j* (*j* = [1…*M*_*tot*_ × *N*_*tot*_], *j* ≠ *i*). **c** Illustration of the pMUT fabrication procedure. **d** (**i**). Top view of the pMUT cell surface and the cross-sectional view of the deposited PZT thin film captured by SEM. (**ii**). Electrode surface roughness measurement results for the pMUT. **e**–**g** Schematic of the acoustic characterization experiments. **e** Transmission characterization. **f** Receiving characterization. **g** Pulse–echo characterization

The bilayer diaphragm pMUT cell is designed to operate in d_31_ flexural-vibration mode (Fig. S1). As shown in Fig. 2a_i, the SOI-based pMUT consists of a ceramic lead zirconate titanate (PZT) layer sandwiched between two thin electrodes, jointly driving the passive Si layer in a cavity to transmit or receive acoustic signals. It is characterized by the electrical capacitance *C*_*0*_, the mechanical impedance *Z*_*m*_, the acoustic impedance *Z*_*a_selfi*_ and the transmit pressure *P*_*out*_. The top electrode is optimized at 67% of the cavity size to maximize the vibration energy^23^. For the circular pMUT cell, the equation for the vibration within a fixed membrane boundary can be derived from a specific form of the Helmholtz equation. The out-of-plane displacement *w(r,t)* of the diaphragm can be expressed as^39^

1 $$D{\nabla }^{2}{\nabla }^{2}w=q-{\rho }_{s}\frac{{\partial }^{2}w}{\partial t}$$

where *q* is the external pressure acting on the surface of the plate, and *ρ*_*s*_ is the mass density per unit area. *D* denotes the plate flexural rigidity. Please refer to the Supplementary Materials and Fig. S1 for the detailed solutions. According to the EQC model shown in Fig. 2b, each cell driven by a voltage *V*_*ac*_ changes the energy flow current *I* in the electrical domain to the volumetric velocity of the vibrated membrane *U* in the mechanical domain, the efficiency of which is characterized by the electromechanical transduction ratio *η*.

In the acoustic domain, *Z*_*a_selfi*_ is the self-acoustic radiation impedance derived from Eq. (1), which can be expressed as follows^40,41^:

2 $${Z}_{a{\rm{\_}}{selfi}}=\frac{\rho c\left[\left(1-\frac{{J}_{1}\left(2k{r}_{{eff}}\right)}{k{r}_{{eff}}}\right)+i\frac{{H}_{1}\left(2k{r}_{{eff}}\right)}{k{r}_{{eff}}}\right]}{A}$$

where *k* is the wavenumber, *A* is the effective area of the diaphragm, *r*_*eff*_ is the effective radius of the pMUT, *J*_*1*_ is the first-order Bessel function of the first kind and *H*_*1*_ is the first-order Struve function.

Next, we connected several cells in parallel to obtain an element driven by a single channel, as shown in Fig. 2a_ii, and extended the element layout to the pMUT array. Because the radiation pressure dominates in imaging applications, we considered acoustic coupling within the array. Specifically, a pMUT cell surrounded by others vibrates not only by its own reaction force but also by the interconnection with its neighbors, namely, the mutual radiation impedance, as shown in Fig. 2a_iii. The presence of mutual radiative impedance replaces the linearity between the cell displacement and the drive voltage, resulting in multiple resonance peaks in the spectrum^26^. The mutual radiation impedance can be expressed as a function of the active element *r*_*eff*_, the distance *d* between two cell centers and the wavenumber *k* as follows:

3 $${Z}_{{ij}}=\frac{9\rho c}{5\pi {{r}_{{eff}}}^{2}}A\left(k{r}_{{eff}}\right)\frac{\sin {kd}+j\cos {kd}}{{kd}}$$

where function *A* can be found by curve fitting using a 10th-order polynomial^42^. We therefore propose the cell–element–array EQC model shown in Fig. 2b. Within each element, the equivalent circuit representation of each element consists of *N*_*_tot*_ pMUT cells driven in parallel by *V*_*ac*_. When considering the pMUT array of *M*_*_tot*_ elements, the acoustic impedance network developed as a 2D matrix *[Z*_*a*_*]*_*ij*_ at the size of *[N*_*_tot*_ *× M*_*_tot*_*, N*_*_tot*_ *× M*_*_tot*_*]* addresses the acoustic coupling between cells. Thereafter, the total acoustical impedance of the *i*^*th*^ pMUT *Z*_*ai*_ is the sum of the self-radiation impedance *Z*_*a_selfi*_ and the mutual radiation impedance *Z*_*ij*_^43^:

4 $${Z}_{{ai}}={Z}_{a{{\_}}{selfi}}+\mathop{\sum }\limits_{\begin{array}{c}j=1\\ i\,\ne\, j\end{array}}^{{M}_{{tot}\times }{N}_{{tot}}}\frac{{U}_{j}}{{U}_{i}}{Z}_{{ij}}$$

In transmission mode, the volumetric velocity of each cell can be calculated by obtaining the KVL equation of the equivalent circuit model^26,27^. If the input voltage is known, the volumetric velocity of the device can be found as

5 $$\begin{array}{l}{U}_{{Num}\times 1}={\left\{{I}_{{Num}\times {Num}}.\times {Z}_{{m}_{{Num}\times 1}}+{Z}_{{a}_{{Num}\times {Num}}}\right\}}^{-1}\\\qquad\qquad\;\;\; \times\,{diag}\left({\eta }_{{Num}\times 1}{V}_{{{ac}}_{1\times {Num}}}\right)\end{array}$$

Then, the sound pressure at any point in space can be calculated by superimposing the sound pressure of all cells at that point^26,43^:

6 $$\begin{array}{c}p\left(r,\theta ,\varphi \right) =\mathop{\sum}\limits_{n=1}^{N}\mathop{\sum}\limits_{m=1}^{M}{p}_{i}\left(m,n\right)\\ \qquad\qquad\qquad\qquad\qquad\qquad\qquad =\mathop{\sum}\limits_{n=1}^{N}\mathop{\sum}\limits_{m=1}^{M}\left(\frac{j\rho {ck}{U}_{i}}{2\pi}\right.\times \frac{{e}^{-{jk}{r}_{i}\left(m,n\right)}}{{r}_{i}\left(m,n\right)}\times {dir}\left(\theta ,\varphi \right)\end{array}$$

where *dir (θ, φ*) is the directivity function of each cell and can be assumed as 1 because *ka* ≤ 1 is valid for most pMUT designs. In addition, *r*_*i*_ *(m, n)* is the distance from an arbitrary point in the medium to pMUT (m, n):

7 $${r}_{i}\left(m,n\right)={\left(\begin{array}{c}{(r\sin \theta \cos \varphi -(m-{m}_{c)}L)}^{2}+\\ ({r\sin \theta \sin \varphi -\left(n-{n}_{c}\right)D)}^{2}+({r\cos \theta )}^{2}\end{array}\right)}^{0.5}$$

where *(m*_*c*_*, n*_*c*_*)* denote the coordinates of the center cell, and *L* and *D* are the pitches between two cells.

During receiving, the received voltage or current of each cell can also be obtained according to the KVL equation of the equivalent circuit model when the incident sound pressure is known^44^:

8 $${V}_{{out}}=\frac{\eta {P}_{{in}}}{j\omega {C}_{0}\times \left({Z}_{m}+{Z}_{i}+{\eta }^{2}\right)}$$

9 $${I}_{{out}}=\frac{\eta {P}_{{in}}}{{Z}_{m}+{Z}_{i}}$$

EQC analysis was implemented in MATLAB R2020a (MathWorks Inc., Natick, MA, USA), and the results were compared with those derived from the finite element method (FEM) in COMSOL Multiphysics v6.0.

### Fabrication process of the 2D MEMS ultrasonic transducer array

As shown in Fig. 2c, each pMUT cell is fabricated based on sputtered Pt/PZT/Pt films on a silicon-on-insulator (SOI) wafer, where a PZT film deposited at 500 °C by DC-pulse reactive magnetron sputtering achieves an average stress of less than 50 MPa. For each pMUT cell, the 5-μm PZT layer, activated by the 0.35-μm top and 0.4-μm bottom Pt layers, drives the 8-μm passive top Si layer together to produce the d_31_ flexural vibration mode. As mentioned above, in the design, the top Pt electrodes are patterned at a size of 67% of the cavity diameter to maximize the output performance (please refer to Fig. S3(C) in the Supplementary Materials for details). Thereafter, the top PECVD silicon nitride (SiNx) layer serves to passivate the PMUT cell and prevent Pt oxidation. Two-step etching (III–IV) causes the surface pad metal, Aurum (Au), to connect the bottom and the top electrodes through opened vias. Finally, the backside DRIE process (VI) is utilized to prepare cavities, defining the effective flexural membrane diameter and boundary conditions. For the pMUT elements, we group the cells in two layouts, namely, 3 × 3 and 4 × 4, with the top electrodes connected and driven by one individual channel. In the pMUT arrays, a fine connection via wires is designed to reach the surrounding array electrodes, including the 3 × 3 and 8 × 8 arrays. Due to the equivalent acoustic impedance between water and soft tissue, a 100-nm layer of perylene was deposited on the surface to make the array compatible with the following in-water experimental environment. Figure 2d_i shows scanning electron microscopy (SEM) images of the surface morphology and a cross-sectional view of the PZT thin films, depicting a vertical crystallographic orientation and verifying the acceptable quality of the film^23^. The roughness parameter Sa of the electrode surface of the pMUT reaches 1.42 nm, as shown in Fig. 2d_ii.

### Experimental setup for electrical, mechanical and acoustic characterization

To analyze the electrical performance, a network analyzer (5230A, Agilent Technologies, Santa Clara, USA) is used to measure the S_11_ parameter and the electrical impedances, whose peak values represent the resonance frequencies of the pMUT elements in both air and water. Using a laser Doppler vibrometer (LDV, MSA600, Polytec GmbH, Germany) to measure the displacement by detecting Doppler shifts from the vibrating surfaces of pMUT membranes, we measured the vibration velocities swept in the frequency spectrum to confirm the resonance frequencies. At the resonance frequency, we used the LDV to measure the vibration of the central point of the target membrane and derived the peak displacement and peak velocity. From an acoustic perspective, a hydrophone Onda HGL-0200 (Onda Corp., CA, USA), a standard transmitter (Olympus Corp., Tokyo, Japan), a pulse generator (33250 A, Agilent Technology, Santa Clara, USA), an oscilloscope (RTO 1002, Rohde & Schwarz, Munich, Germany) and a three-axis stage (DTS50, Thorlabs) were built together as planforms to determine the transmission (Fig. 2e), reception (Fig. 2f) and pulse–-echo (Fig. 2g) performance levels.

### Experimental setup for ultrasound imaging using the pMUT array transducer

Data acquisition was performed using the Ultra-DAQ-256 ultrasound system (Ultra-DAQ-256, Hisky Med, Beijing, China), which provides an analog front-end sufficient to fully control the transmit and receive components up to 256 elements. To interface the fabricated pMUT array transducers with the Ultra-DAQ-256 system, an adaptable connector was designed and built to ensure compatibility with a universal 64-channel transducer adapter. There is no electrical impedance matching design for the pMUT array transducer with the Ultra-DAQ-256 system. The measured transducer impedance was 50 Ω, and the internal impedance matching of the ultrasound system was set to 50 Ω. In all the experiments, the pMUT array transducer was connected to the Ultra-DAQ-256 system using a custom connector and was driven with a 5-cycle 5-V sine pulse at a 3-MHz center frequency.

As a first step toward imaging characterization, we built a wire phantom consisting of three hairs within one 2D plane whose average diameter is 0.05 mm (Fig. 6a). All hairs were submerged in deionized water to a depth of 20 mm. In the experiment, the pMUT array was positioned at the bottom of the water tank to acquire 3D volumetric ultrasound images of the hairs using the Ultra-DAQ-256 system.

Next, the vascular ultrasound phantom was built for the performance assessment of the phased array. To mimic the realistic imaging properties of the vasculature^45^, a homogeneous phantom was prepared by mixing 10% (by weight) polyvinyl alcohol (PVA), 87% distilled water and 3% sigma cell cellulose as ultrasound scattering particles at a temperature of 85 °C. The mixture was thereafter poured into a 3D-printed mold to produce a realistic geometry, and the materials were placed together in a freezer. After two freeze–thaw cycles, a vascular phantom with an axial length of 36 mm, an inner diameter of 7 mm and an outer diameter of 14 mm was obtained (Fig. 7a_i).

## Results and discussion

### Electrical, mechanical, and acoustic characterization of the MEMS ultrasonic phased-array transducer

To analyze a single cell, the results of the EQC model were compared with those of the corresponding FEM models. The proposed pMUT is an SOI structure consisting of an 8-μm thick Si passive layer and a 5-μm thick PZT piezoelectric layer sandwiched between the top and bottom electrodes with a 100-μm radius. The structural and material properties of a single circular pMUT are listed in Table S2. In the EQC model, the volumetric displacement–frequency responses in air and water are simulated and suitably agree with the FEM model results. Please refer to the Supplementary Materials for details.

**Fig. 3:**
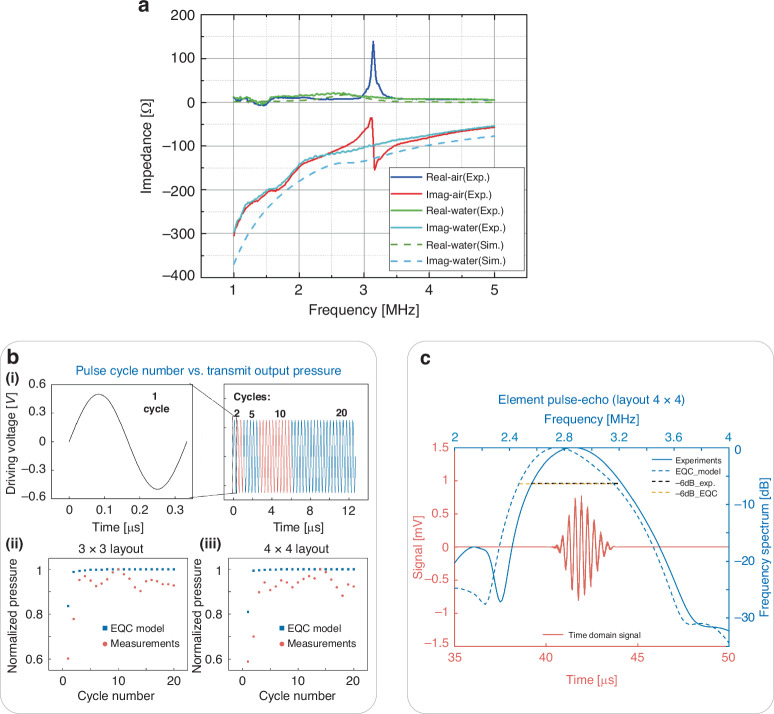
Electrical impedance, linear response, and pulse-echo performance measurements of pMUT. **a** The electrical impedance of one element is characterized via simulations and experiments. **b** Optimization study of the number of pulse cycles to maximize the transmit output pressure for a single element. (**i**). Waveforms of the actuated pulse when the cycle number is 2, 5, 10 and 20. (**ii**). Influence of the number of pulse cycles on the output pressure in the 3 × 3 layout of one element. (**iii**). Influence of the number of pulse cycles on the output pressure in the 4 × 4 layout of one element. **c** Bandwidth (BW) characterization of a single element using pulse–echo signals by equivalent circuit model (EQC) simulations and experiments

Each element driven by one channel is designed by grouping multiple cells in parallel, aiming to reduce the channel number for the high-density pMUT phased array. In this study, we assessed the performance of elements in two layouts, one with 9 cells (3 × 3 layout) and the other with 16 cells (4 × 4 layout), in which the cell pitch was normalized to 250 μm. First, the electrical impedance in air measured experimentally peaked at 3.2 MHz, while the in-water impedance reached 2.8 MHz in both the FEM and experimental results (Fig. 3a) due to the load change. Considering this minor difference, the resonance frequencies in the following in-air and in-water characterizations were set to 3 MHz. Next, we optimized the number of pulse cycles to maximize the pressure output (Fig. 3b). After sweeping from 1 to 20 cycles, we found that a 5-cycle pulse could provide the maximum pressure output in the experiment, which is consistent with the EQC results. Therefore, the following examination entailed the use of 5 cycles of pulses for actuation. To characterize the pulse-echo properties, we measured the pulse-echo signal from the steel plate 35 mm away from the pMUT surface in the water tank (Fig. 3c). After applying a Hanning window at the received signal, we obtained a -6 dB bandwidth (BW) of 25.5% centered at a 2.9-MHz resonance frequency, which is consistent with the EQC model prediction (25.23% BW centered at 2.7 MHz). A frequency shift of approximately 0.2 MHz occurred between the EQC results and the experimental measurements. This shift is likely due to the fact that the EQC model calculates only the vibrations of the active and passive layers. In addition, the initial stress introduced by pMUT fabrication, which may cause a frequency shift, was not included at this moment. The measured in-water resonance frequencies also agreed well with those obtained in the electrical impedance measurements (Fig. 3a). Moreover, we addressed the possibility of increasing the bandwidth for the pMUT array, which could be achieved by placing cells of different sizes together in a fluid acoustic medium and utilizing the large damping effect of the acoustic medium to merge the multiple resonance peaks to achieve an increased bandwidth^46^. Similarly, the development of rectangular pMUTs with a high aspect ratio has enabled several resonance modes, resulting in an ultrawide merged bandwidth when operated in a large damping medium^47^. Alternatively, the bandwidth could be enlarged by incorporating a polydimethylsiloxane (PDMS) backing structure into pMUTs^48^. However, it is important to note that these approaches may entail a reduction in the effective receiving area within each frequency band, thereby potentially necessitating a trade-off between the sensitivity and bandwidth.

**Fig. 4:**
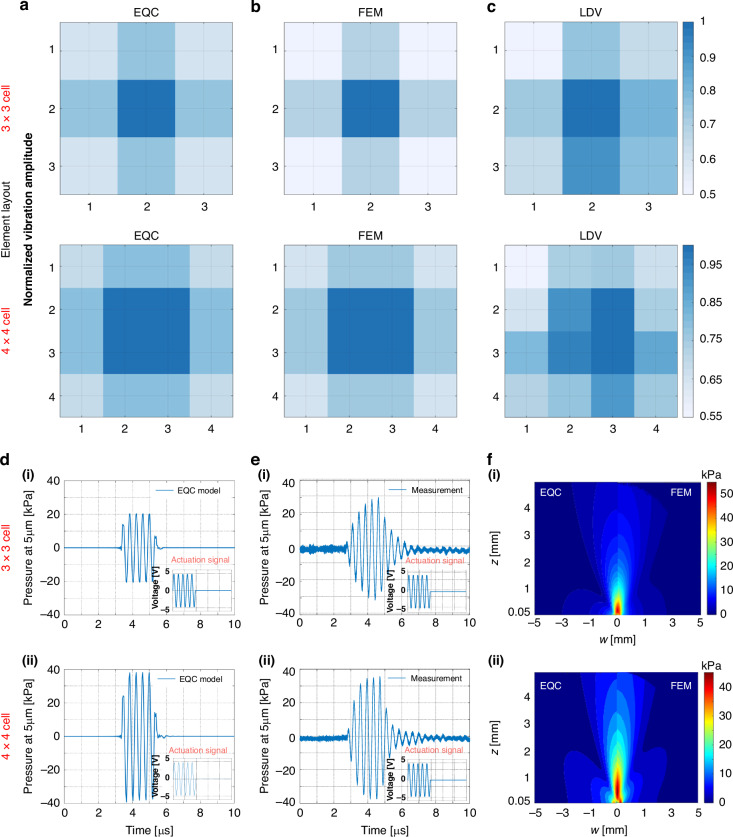
The vibration velocity and two-dimensional pressure field distribution of the single element. **a**–**c** Normalized vibration velocities of all cells within one single element characterized by equivalent circuit model (EQC) simulations, COMSOL simulations and laser Doppler velocimetry (LDV) measurements. **a** Equivalent circuit model (EQC) simulation results. **b** Finite element method (FEM) simulation results. **c** Laser Doppler velocimetry (LDV) measurements. **d** Acoustic transmission efficiency characterized by equivalent circuit model (EQC) simulations. **e** Acoustic transmission efficiency characterized by hydrophone experiments. **f** Comparison of the 2D pressure field between the equivalent circuit model (EQC) and finite element method (FEM) simulations

To characterize the mechanical vibrations within a single element, we used the EQC model to calculate the peak velocities at a 3-MHz resonance frequency of all cells within one element (Fig. 4a–c), considering the mutual acoustic coupling effects. The EQC model-derived results were compared with the FEM references as well as the experimental counterparts measured by the LDV. The normalized velocities within the 3 × 3-layer and 4 × 4-layer elements matched well, especially for the EQC model and the FEM model, indicating the validity of the EQC model. The results of the LDV experiments are slightly different, as shown in Fig. 4c, and are not as symmetrical as those shown in Fig. 4a, b. In the simulations, we accounted for interference through the acoustic medium and focused on calculating the vibration of the dominant active and passive layers to manage the analytical approximations and computational complexity. However, in the LDV measurements depicted in Fig. 4c, environmental factors may introduce electrical and mechanical crosstalk phenomena, as well as vibrations from the substrate and surroundings, resulting in these asymmetrically distributed results. Nevertheless, both the LDV measurements and simulation results exhibited similar trends, with peak values concentrated at the center.

In acoustic output characterization of one single element, the axial pressures at 5 mm from the pMUT surface evaluated by the EQC model (Fig. 4d_i and D_ii) are consistent with the reference data acquired by hydrophone experiments (Fig. 4e_i and e_ii). The simulated waveforms resemble more closely those of the 5-cycle actuation pulses, while the measured results are more realistic because of the full damping effects of the PZT membrane. The overall 2D pressure fields simulated by the EQC model agree with those simulated by the FEM model for both the 3 × 3 layout (Fig. 4f_i) and 4 × 4 layout (Fig. 4f_ii) within one element.

In Table 1, we summarize the acoustic characterizations of the 3 × 3 layout and 4 × 4 layout for a single element. In general, the EQC model can be used to characterize the transmit efficiency at a 5-mm distance and the receive sensitivity in terms of the voltage and current, demonstrating agreement with the FEM and experimental results.

**Table 1:** Results of the transmission and receiving experiments of a single element

	Transmit: *P*_out_ (at 5 mm) [kPa/V]		Receive: *V*_out_ [mV/kPa]		Receive: *I*_out_ [μA/kPa]	
Layout	3 × 3	4 × 4	3 × 3	4 × 4	3 × 3	4 × 4
EQC Model	4.24	7.75	4.69	3.83	17.09	29.25
FEM Model	5.6	10	4.3	4.4	18	31
Experiments	6.14	7.32	N/A	N/A	N/A	N/A

**Fig. 5:**
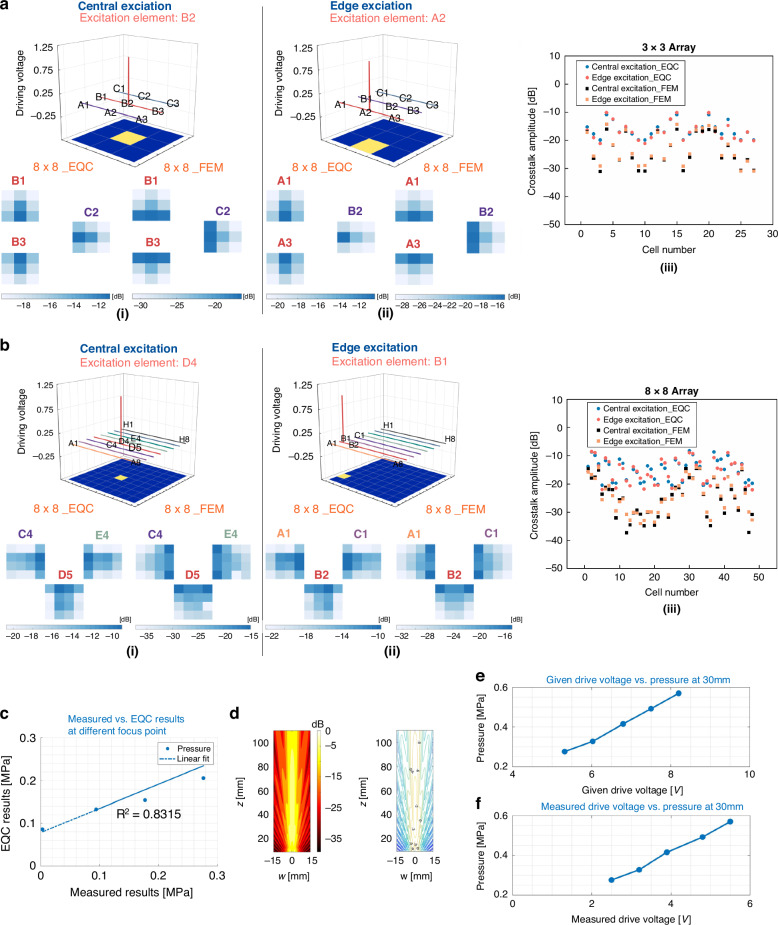
Quantitative crosstalk analysis, focused pressure measurement, and two-dimensional acoustic field distribution of pMUT arrays. **a**, **b** Acoustic coupling effects from central excitation and edge excitation characterized by the equivalent circuit model (EQC) model and the FEM model. **a** Cross-talk analysis of the 3 × 3 array. (**i**) An array using central excitation at element D4, in which neighboring elements B1, B3, and C3 are characterized. (**ii**) An array using edge excitation at element A2, in which neighboring elements A1, A3, and B2 are characterized. (**iii**) Quantitative analysis results for the cross-talk degree (in dB) using the equivalent circuit model (EQC) model and the FEM model. **b** Cross-talk analysis of the 8 × 8 array. (**i**) An array actuated with central excitation D4, in which neighboring elements C4, E4, and D5 are characterized. (**ii**) An array actuated with edge excitation B1, in which neighboring elements A1, C1, and B2 are characterized. (**iii**). Quantitative analysis results for the cross-talk degree (in dB) using the equivalent circuit model (EQC) model and the FEM model. **c**–**f** Varied focusing intensities of the 8 × 8 MEMS phased-array transducer characterized by equivalent circuit model (EQC) simulations and experimental measurements. **c** Focused pressure at different depths: Comparison between the calculated results of the equivalent circuit (EQC) model and the experimental measurements. **d** Spatial pressure field distribution of the entire array calculated by the equivalent circuit (EQC) model. **e** Relationship between the excitation voltage amplitude set in the imaging platform and the focused pressure measured at 30 mm. **f** Relationship between the actual voltage amplitude on the device and the focused pressure measured at 30 mm

To verify the uniformity across the 8 × 8 pMUT phased array, we calculated the center frequencies of all 64 elements from electrical impedance measurements. The 8 × 8 pMUT phased array is indeed centered at 3 MHz with a minor deviation of <3.2%, as prescribed (Fig. S3). Moreover, the cross-talk phenomenon, which is representative of acoustic coupling, can be characterized by the EQC and FEM models for the 3 × 3 pMUT phased array and the 8 × 8 pMUT phased array (Fig. 5a, b). For the small array of 3 × 3 elements, the cross-talk distribution is more symmetric in all orientations regardless of the central excitation (actuating element B2, Fig. 5a_i) or edge excitation (actuating element A2, Fig. 5a_ii). For the large array of 8 × 8 elements, the cross-talk phenomenon exhibits high dependence on the location of the actuated elements (central excitation Fig. 5b_i vs. edge excitation Fig. 5b_ii). As shown in Fig. 5a_iii and b_iii, the cross-talk level of all cells simulated by the EQC model follows that derived from the FEM model, especially for the 8 × 8 array. Notably, there is an overestimation of the cross-talk amplitude in the EQC model compared to that in the FEM results. The cross-talk levels of the 3 × 3 pMUT array and the 8 × 8 pMUT array can reach as low as -32 dB and -35 dB, respectively, which can contribute to improved imaging resolutions for pMUT phased-array transducers.

To examine the focusing properties of the 8 × 8 pMUT array, we set the phase delay of 64 elements and measured the pressure at focusing depths of 22 mm, 29 mm, 47 mm and 69 mm when the 5-V actuation voltage remained fixed. In Fig. 5c, the focused pressure intensities are underestimated by the EQC model, but the simulated trend follows that in the experimental measurements. When the focus depth is fixed at 30 mm, the measured pressure intensity increases linearly with drive voltage (from 5 V to 10 V), as shown in Fig. 5e, f.

### In vitro 3D volumetric imaging of the hair phantom and vascular phantom

**Fig. 6:**
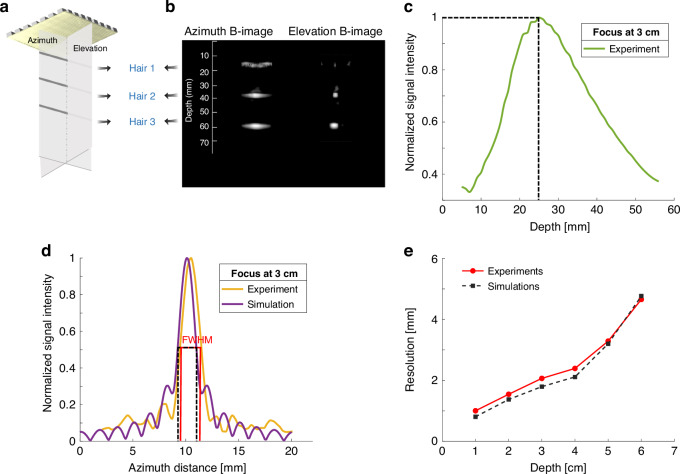
Hair imaging experiments and lateral resolution measurements of 8×8 pMUT array. **a** Illustration of the hair phantom imaging setup. **b** Three-dimensional volumetric imaging results of the 8 × 8 pMUT phased-array transducer. **c** Measured pressure intensity with axial distance in front of the pMUT phased-array transducer when focused at 3 cm. **d** Measured pressure intensity with lateral distance when focused at 3 cm, where a −3 dB beamwidth is defined as the lateral resolution. **e** Measured lateral resolutions (marked in red) of the pMUT array at different depths compared to the simulated results (marked in black)

To evaluate the imaging properties of the 8 × 8 pMUT phased array, we first performed plane wave actuation of 64 elements and utilized a generalized coherence factor (GCF)-based adaptive beamforming method^49^ for receiving 3D volumetric images of the three-wire phantom in water (Fig. 6a). The 3D volumetric image was projected into two orthogonal planes, as shown in Fig. 6b. As such, the azimuth B-image shows three hairs located at depths of 15 mm, 35 mm and 55 mm, while the elevation B-image shows the cross-sections of these three hairs. In the elevation B-image, the later beam profiles are derived at depths of 15 mm, 35 mm and 55 mm.

To quantitatively evaluate the imaging performance, lateral resolutions at different depths were investigated. In Fig. 6c, upon programming the multichannel phased delays to focus at 3 cm, the measured sound pressure peaked near the axial distance of 2.6 cm and confirmed the validity of the focusing approach. Within the fixed axial distance of 3 cm, Fig. 6D shows the measured beam profile across the lateral dimension in yellow, with the lateral resolution defined based on the full width at half maximum, resulting in a lateral resolution of 2 mm. Subsequently, we evaluated the lateral resolution at various depths and compared it with the simulation results of the EQC model. Figure 6e shows that the simulation results suitably agree with the experimental results. Specifically, the minimum resolutions are approximately 1 mm at a depth of 1 cm, 1.5 mm at a depth of 2 cm, and 2.4 mm, 3.3 mm, and 4.6 mm at depths of 4–6 cm.

**Fig. 7:**
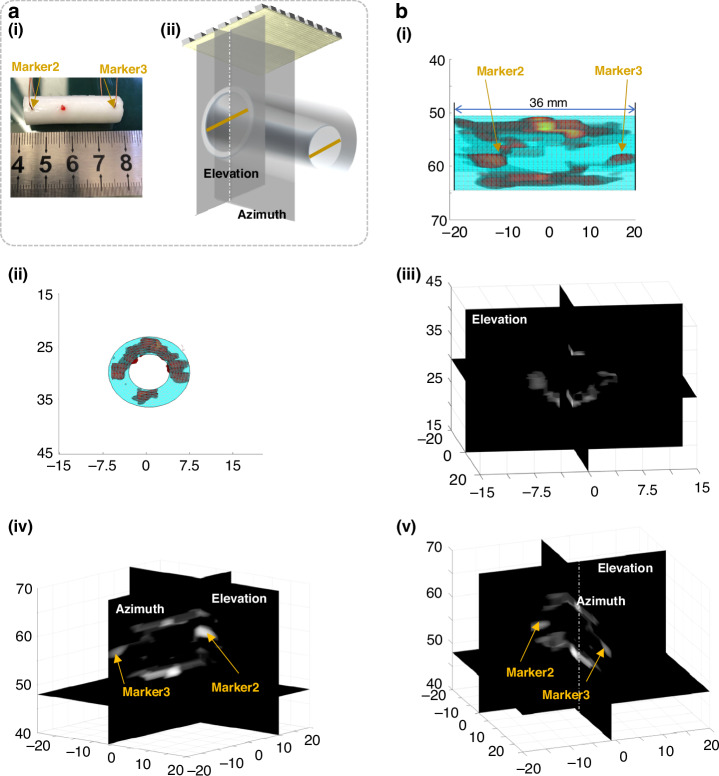
Vascular phantom experiments with 8×8 pMUT array. **a** Illustration of the vascular phantom (i) and the built-up image (ii). **b** Contoured 3D volumetric vascular imaging results at depths of 55 mm and 30 mm (i-ii) and their 2D orthogonal projections using an 8 × 8 pMUT phased-array transducer (iii-v)

To investigate the applications of in vivo vascular imaging, we investigated the performance of the 8 × 8 pMUT phased array in reconstructing 3D volumetric images of the vascular phantom (Fig. 7a). The vascular phantom exhibits a length of approximately 36 mm and a diameter of approximately 14 mm. Two line markers were positioned at each end of the vascular phantom at a distance of 30 mm. During imaging, the phantom was positioned over the pMUT at distances of approximately 55 mm and 30 mm from the surface of the device. In Fig. 7b_i, the 3D image covering the volumetric space (azimuth × elevation × depth) of 40 mm × 40 mm × 70 mm shows the profile of the vasculature together with wire markers (denoted as Markers 2 and 3). Based on the 3D imaging results, the reconstructed phantom profiles were contoured at 36 mm with the two markers positioned 30 mm apart, as shown in Fig. 7b_i, which is consistent with the measurements depicted in Fig. 7a_i. In Fig. 7b_iv, the 3D volumetric image data are projected into two orthogonal planes, including an annular cross-sectional view with Marker 2 (in the azimuth B-image) and an axial view of the upper and lower vascular walls (in the elevation B-image). Subsequently, we adjusted the imaging depth to 3 cm to illustrate the variance. As shown in Fig. 7b_ii-iii, the circular profile with a diameter of 1 cm at a depth of 3 cm can be characterized, agreeing with the phantom setup. In this study, the frame rate, i.e., the temporal resolution, can reach as high as 11 kHz, which is sufficient for fast imaging of 3D volumetric vascular dynamics.

## Discussion

### Importance of EQC analysis for pMUT-based 3D imaging

For traditional array transducers, the pitch, defined as the element spacing between different elements, is expected to remain within half a wavelength range to suppress artifacts caused by grating lobes and to reduce blurring effects at the sensing target^50,51^. Increasing the number of elements can amplify the aperture size, narrowing the beam and improving the lateral resolution during imaging^50^. Multichannel pMUT arrays can benefit from small cells and their high-density distribution to achieve the desired energy output for a high signal-to-noise ratio. However, actuating each cell via an individual channel remains challenging for drive circuits. Therefore, grouping multiple cells into one single element/channel is normally utilized to achieve a high output for large pMUT arrays. However, the grouped cells within each element increase the size of the pitch, resulting in the abovementioned artifacts. When considering grouping pMUTs for large-array design, it is crucial to acknowledge the trade-off between the number of cells and the element spacing to enhance the imaging performance. The proposed EQC analysis facilitates quantitative evaluation of this prevalent design framework for programmable large pMUT arrays, thereby enabling the enhancement in imaging properties through optimized pMUT arrangements.

### Accuracy assessment of the EQC model for phased-array pMUT transducers

As shown in Fig. 3c, the presence of residual stresses, related to the processing conditions of each layer, affects the initial shape and stiffness of the membrane, which in turn affects the fundamental intrinsic frequency of pMUTs^52^. Based on the resonance frequency measured by the LDV, we can fit the EQC model-derived results to approximate the residual stresses in the membrane vibration equations. Normally, the residual stress introduced by the same fabrication process probably remains within a certain range. Therefore, we can predict the resonance frequency performance for new designs by including residual stresses in the improved EQC models.

As indicated in Table 1, the acoustic results derived from the EQC model and FEM simulations deviate more notably than do the vibration displacement results. In the EQC model, we utilized a far-field approximation to calculate the pressure, while in the FEM, we approximated each grid as a point source. As a result, the difference increases when calculating the near-field pressure (at 5 mm) for a small number of pMUT cells within one element. However, the difference can be minimized for assessing the 7-cm depth imaging performance of the whole array, as shown in Fig. 6d.

### Prospects for future pMUT-based 3D in vivo imaging

As shown in Fig. 6e, the achieved minimum lateral resolution at a depth of 1 cm was approximately 1 mm. Consequently, the smallest size of biological tissue observable at a depth of 1 cm is approximately 1 mm. Based on tissue or organ dimensions at various depths reported in the literature (Fig. S6)^8^, vasculatures together with biological structures such as skeletal muscle and diaphragm could be observed within the resolution and penetration depth capabilities of our device. The resolution of the pMUT array is comparable to that reported by Hu at depths of 4 cm and below^9^, and it is similar to the minimum spatial resolution of the commercial butterfly iQ probe (Butterfly Network, Inc., Guilford, CT)^53^. The butterfly iQ probe has been demonstrated effective in point-of-care ultrasound (POCUS) applications^54^. Consequently, we believe that POCUS diagnoses within a 7-cm depth, such as arterial hypotension, shock and soft tissue infections, may also fall within the detection range of our device.

We would like to address the three main challenges encountered when advancing our low-voltage-driven pMUT array toward realistic 3D volumetric imaging.

1)In contrast to conventional bulk PZT transducers, pMUT arrays operate in a thin-film vibration format with low electrical impedance. Therefore, it is crucial to develop effective electrical and acoustic coupling methodologies to maximize pulse–echo signals. Our proposed EQC model offers a solution by directly incorporating electrical impedance and acoustic impedance values, allowing for numerical evaluation of impedance matching.

2)Traditional PCB drive methods pose challenges in fitting into high-density pMUT arrays. As mentioned in the section of introduction, CMOS circuits fabricated using compatible processing technology with pMUTs could enhance the multichannel transmit/receive performance in arrays. However, the development of pMUT-based CMOS design is still at its early stages. Moving forward, integrating the developed EQC model with CMOS design can facilitate full optimization.

3)Finally, CMOS-integrated pMUT arrays require novel multichannel beamforming algorithms to enhance the 3D volumetric imaging results in a new format. The proposed methodologies can thereafter include the development of advanced signal processing techniques, specifically for pMUT arrays integrated with CMOS technology.

## Conclusion

The low-voltage-driven MEMS ultrasonic phased-array transducer proposed in this study enables noninvasive 3D volumetric imaging with a large penetration depth and high temporal resolution. With the use of a multilevel cell–element–array design and a MEMS-based fabrication approach, this study reports a 5-V-actuated 8 × 8 phased-array transducer for 3D volumetric imaging of a vascular phantom (40 mm azimuth × 40 mm elevation × 70 mm depth) at a temporal resolution of 11 kHz. Low-voltage-driven fast 3D volumetric imaging of the vasculature can facilitate long-term monitoring of hemodynamics and vascular proliferation in deep tissue to benefit various conditions and diseases. Monitoring and imaging the full dimensions of blood vessels at a high speed is important for evaluating vessel functions and diagnosing and treating vascular diseases. For example, capturing volumetric hemodynamic changes in response to vascular occlusion can assist in examining venous compliance, a key indicator of cardiac function^55,56^.

Although the low-voltage-driven pMUT phased-array transducer described here only aims to image 3D vascular phantoms, this platform technology can potentially be extended to monitor many other organs in deep tissues, such as bladders, kidneys, and lungs. Furthermore, developing beamforming methods adapted to the pMUT transducer can further improve the signal intensity and increase the penetration depth. Moreover, multiple resonance frequencies can achieve a notable imaging depth while maintaining a high spatial resolution. It has been demonstrated that pMUTs can achieve multiple resonance frequencies by controlling their vibration profile^57–61^. It is possible to integrate two or more resonance frequencies in the proposed approach to improve the spatial resolution. Additionally, we can group multiple pMUT arrays to increase the aperture number for high-signal-to-noise ratio (SNR) imaging at an enlarged 3D volume considering their easy MEMS-based fabrication. Finally, it is easy to integrate this 5-V-driven pMUT array with multichannel CMOS integrated circuits due to the use of the same processing technologies, paving the way for wearable ultrasound-on-chip platforms.

## Supplementary information

Supplemental Material
