# Supplementary material for: A low-voltage-driven MEMS ultrasonic phased-array transducer for fast 3D volumetric imaging
Source: Microsyst Nanoeng. 2024 Sep 12;10:128. doi: 10.1038/s41378-024-00755-9 (PMC11391059; doi:10.1038/s41378-024-00755-9)
Supplement: Supplementary file 1 — Supplemental Material [file 41378_2024_755_MOESM1_ESM.docx]

## Supplementary Information for

**A low-voltage-driven MEMS ultrasonic phased-array transducer for fast 3D volumetric imaging**

Yun Zhang^1,2^, Tong Jin^1,2^, Yining Deng^1,2^, Zijie Zhao^1, *^, Rui Wang^3^, Qiong He^3^, Jianwen Luo^3^, Jiawei Li^4^, Kang Du^4^, Tao Wu^4^, Chenfang Yan^1,2^, Hao Zhang^1^, Xinchao Lu^1^, Chengjun Huang^1,2, *^, and Hang Gao^1, *^

1 Institute of Microelectronics of the Chinese Academy of Sciences, Beijing, 100029, China

2 University of Chinese Academy of Sciences, Beijing, 100049, China

3 School of Biomedical Engineering, Tsinghua University, Beijing, 100084, China

4 School of Information Science and Technology, ShanghaiTech University, Shanghai, 201210, China

## Single-cell equivalent circuit (EQC) model

A single cell in the array is developed based on a silicon-on-insulator (SOI) structure, which has a stack of several layers. A schematic diaphragm of the unimorph piezoelectric micromachined ultrasound transducer (pMUT) is shown in Fig. S1A. The pMUT contains a layer of piezoelectric material, which is sandwiched between two thin electrodes Since the pMUT relies on the strain mismatch between the active and passive layers to generate vibrations and to transmit or receive acoustic signals, we consider two main layers in the equivalent circuit model, the active layer (piezoelectric layer, PZT) and the passive layer (Si). The bottom face of the silicon layer is at z =0 as shown in Fig. S1B and the out-of-plane displacement of the plate is in the negative direction along the z-axis, which is denoted by *w(r,t)*. Since the diameter of a circular pMUT is much larger than its thickness, the equivalent circuit (EQC) model can be settled based on classic plate theory[^39^](#_ENREF_28).

For such a multi-layer structure, it has a neutral layer:

$$\begin{aligned} z_{m}=\frac{\sum_{i=1}^{n} Y_{i}^{'}z_{i}h_{i}}{\sum_{i=1}^{n} Y_{i}^{'}h_{i}}\#\left( AUTONUM \backslash* Arabic \right) \end{aligned}$$

$$\begin{aligned} Y_{i}^{'}=\frac{Y_{i}}{1-v_{i}^{2}}\#\left( 2 \right) \end{aligned}$$

where *Y_i_*, *v_i_*, *h_i_* and *z_i_* are Young’s modulus, Poisson’s ratio, thickness and the middle position of the *i*^th^ layer.

The vibration equation of the circular diaphragm is[^39^](#_ENREF_28):

$$\begin{aligned} D\nabla^{2}\nabla^{2}w=q-\rho_{s}\frac{\partial^{2}w}{\partial t}\#\left( 3 \right) \end{aligned}$$

where *q* is the external pressure acting on the surface of the plate, and the mass density per unit area *ρ_s_* is the density multiplied by the thickness. *D* is the plate flexural rigidity and can be presented as follows[^39^](#_ENREF_39):

$$\begin{aligned} D= \sum_{I=1}^{n} Y_{i}^{'}\left[ I_{i}+Z_{i}^{2}h_{i} \right]\#\left( 4 \right) \end{aligned}$$

The second moment of inertia and the distance from the neutral axis *z_m_* to the center of the *i*th layer are denoted as *I_i_* and *Z_i_*:

$$\begin{aligned} I_{i}=\frac{h_{i}^{3}}{12}\#\left( 5 \right) \end{aligned}$$

$$\begin{aligned} Z_{i}=z_{i}-z_{m}\#\left( 6 \right) \end{aligned}$$

In case of simple harmonic excitation, the displacement *w (r, t)* is given by[^39^](#_ENREF_28):

$$\begin{aligned} w=W\left( r \right)e^{-j\omega t}\#\left( 7 \right) \end{aligned}$$

where *ω* is the angular frequency and *W(r)* is the shape function of the circular diaphragm when vibrating. Combining (4) and (7), the simplified equation of motion for the membrane vibration can be obtained:

$$\begin{aligned} \nabla^{2}\nabla^{2}W+\beta^{4}W=\frac{q}{D}\#\left( 8 \right) \end{aligned}$$

The general solution of (8) is as follows[^6^](#_ENREF_40)^0^:

$$\begin{aligned} W\left( r \right)=-\frac{q}{\omega^{2}\rho_{s}}+A_{i}\times J_{0}\left( \beta r \right)+B_{i}\times I_{0}\left( \beta r \right)+ \\ C_{i}\times Y_{0}\left( \beta r \right)+D_{i}\times K_{0}\left( \beta r \right),i\in N\#\left( 9 \right) \end{aligned}$$

where *β* is the function of frequency, flexural rigidity and surface density and in the following form:

$$\begin{aligned} \beta^{4}=\frac{\omega^{2}\rho_{s}}{D}\#\left( 10 \right) \end{aligned}$$

and *J_0_(x)*, *I_0_(x)*, *Y_0_(x)* and *K_0_(x)* are the zero-order Bessel function of the first kind, the modified Bessel function of the first kind, the Bessel function of the second kind, and the modified Bessel function of the second kind, respectively. The coefficients *Ai*, *Bi*, *Ci* and *Di* are constants which are determined by boundary conditions.

The shape functions of region I and region II are the forms as follows:

$$\begin{aligned} W_{I}\left( r \right)=-\frac{q}{\omega^{2}\rho_{s}}+A_{1}J_{0}\left( \beta r \right)+B_{1}I_{0}\left( \beta r \right), r\in(0,a)\#\left( 11 \right) \end{aligned}$$

$$\begin{aligned} W_{II}\left( r \right)=-\frac{q}{\omega^{2}\rho_{s}}+A_{2}J_{0}\left( \beta r \right)+B_{2}I_{0}\left( \beta r \right)+ \\ C_{2}Y_{0}\left( \beta r \right)+D_{2}K_{0}\left( \beta r \right), r\in(a,b)\#\left( 12 \right) \end{aligned}$$

As *x* tends to zero, the Bessel function *Y_0_(x)* and *K_0_(x)* approach infinity, so these two Bessel functions are discarded for the calculation of Region I. In order to solve (11) and (12), certain boundary conditions must be satisfied, i.e., continuity and the clamped boundary conditions.

At the junction of Region I and Region II, the displacements of the membrane, the first order derivatives of the displacements, the radial moment and the radial shear forces must be equal to ensure continuity:

$$\begin{aligned} W_{I}\left( a \right)=W_{II}\left( a \right)\#\left( 13 \right) \end{aligned}$$

$$\begin{aligned} \left. \frac{dW_{I}}{dr} \right|_{r=a}=\left. \frac{dW_{II}}{dr} \right|_{r=a}\#\left( 14 \right) \end{aligned}$$

$$\begin{aligned} \left. M_{rr,I} \right|_{r=a}=\left. M_{rr,II} \right|_{r=a}\#\left( 15 \right) \end{aligned}$$

$$\begin{aligned} \left. Q_{r,I} \right|_{r=a}= \left. Q_{r,II} \right|_{r=a}\#\left( 16 \right) \end{aligned}$$

where the radial moments are different in the two regions because only Region I is located directly below the top electrode[^39^](#_ENREF_28):

$$\begin{aligned} M_{rr,I}=-D\left[ \frac{\partial^{2}w}{\partial r^{2}}+\frac{v}{r}\frac{\partial w}{\partial r} \right]-\frac{Y_{0}d_{31}hV}{1-v}\#\left( 17a \right) \end{aligned}$$

$$\begin{aligned} M_{rr,II}=-D\left[ \frac{\partial^{2}w}{\partial r^{2}}+\frac{v}{r}\frac{\partial w}{\partial r} \right]\#\left( 17b \right) \end{aligned}$$

and the radial shear force is defined as[^6^](#_ENREF_40)^0^:

$$\begin{aligned} Q_{r}=-D\left[ \frac{1}{r}\frac{\partial}{\partial r}\left( r\frac{\partial w}{\partial r} \right) \right]\#\left( 18 \right) \end{aligned}$$

For a circular membrane with the clamped boundary conditions, the displacement of the membrane at the edge (r=b) must be zero, and the slope of the displacement along the radial direction is also zero at the edge[^39^](#_ENREF_28):

$$\begin{aligned} W_{II}\left( b \right)=0\#\left( 19 \right) \end{aligned}$$

$$\begin{aligned} \left. \frac{dW_{II}}{dr} \right|_{r=b}=0\#\left( 20 \right) \end{aligned}$$

On the basis of the shape function, the volumetric displacement of the membrane vibration is integrated by the displacement function over the area of the circular membrane[^27^](#_ENREF_25):

$$\begin{aligned} W_{vol}=2\pi\left[ \int_{0}^{a} W_{I}\left( r \right)rdr+\int_{a}^{b} W_{II}\left( r \right)rdr \right]\#\left( 21 \right) \end{aligned}$$

In order to obtain the specific displacement shape function for the region I and region II, it is necessary to use the continuity conditions and the clamped boundary conditions to obtain *A_1_*, *B_1_*, *A_2_*, *B_2_*, *C_2_* and *D_2_*. The boundary conditions (13) - (16) and (19) - (20) lead to six equations to solve for these constants, which are expressed in matrix form[^39^](#_ENREF_28):

$$\begin{aligned} &\left[ \begin{matrix} J_{0}\left( \beta a \right) & I_{0}\left( \beta a \right) & -J_{0}\left( \beta a \right) & -I_{0}\left( \beta a \right) & -Y_{0}\left( \beta a \right) & -K_{0}\left( \beta a \right) \\ J_{1}\left( \beta a \right) & {-I}_{1}\left( \beta a \right) & {-J}_{1}\left( \beta a \right) & I_{1}\left( \beta a \right) & {-Y}_{1}\left( \beta a \right) & {-K}_{1}\left( \beta a \right) \\ JJ\left( \beta a \right) & II\left( \beta a \right) & -JJ\left( \beta a \right) & -II\left( \beta a \right) & -YY\left( \beta a \right) & -KK\left( \beta a \right) \\ JJJ\left( \beta a \right) & III\left( \beta a \right) & -JJJ\left( \beta a \right) & -III\left( \beta a \right) & -YYY\left( \beta a \right) & -KKK\left( \beta a \right) \\ 0 & 0 & J_{0}\left( \beta b \right) & I_{0}\left( \beta b \right) & Y_{0}\left( \beta b \right) & K_{0}\left( \beta b \right) \\ 0 & 0 & -J_{1}\left( \beta b \right) & I_{1}\left( \beta b \right) & -Y_{1}\left( \beta b \right) & -K_{1}\left( \beta b \right) \end{matrix} \right]\# \\ &\times\left[ \begin{matrix} A_{1} \\ B_{1} \\ A_{2} \\ B_{2} \\ C_{2} \\ D_{2} \end{matrix} \right]=\left[ \begin{matrix} 0 \\ 0 \\ \frac{-Y_{0}d_{31}hV}{D\beta^{2}\left( 1-v \right)} \\ 0 \\ \frac{q}{\omega^{2}\rho_{s}} \\ 0 \end{matrix} \right]\#(22) \end{aligned}$$

where the functions *II(x)*, *JJ(x)*, *YY(x)*, *KK(x)*, *JJJ(x)*, *III(x)*, *YYY(x)* and *KKK(x)* are:

$$\begin{aligned} JJ\left( x \right)=-0.5J_{0}\left( x \right)-\left( v/x \right)J_{1}\left( x \right)+0.5J_{2}\left( x \right)\#\left( 23 \right) \end{aligned}$$

$$\begin{aligned} II\left( x \right)=0.5I_{0}\left( x \right)+\left( v/x \right)I_{1}\left( x \right)+0.5I_{2}(x)\#\left( 24 \right) \end{aligned}$$

$$\begin{aligned} YY\left( x \right)=-0.5Y_{0}\left( x \right)-\left( v/x \right)Y_{1}\left( x \right)+0.5Y_{2}\left( x \right)\#\left( 25 \right) \end{aligned}$$

$$\begin{aligned} KK\left( x \right)=0.5K_{0}\left( x \right)-\left( v/x \right)K_{1}\left( x \right)+0.5K_{2}\left( x \right)\#\left( 26 \right) \end{aligned}$$

$$\begin{aligned} JJJ\left( x \right)=-2xJ_{0}\left( x \right)+\left( 4+3x^{2} \right)J_{1}\left( x \right)+2xJ_{2}\left( x \right)-x^{2}J_{3}\left( x \right)\#\left( 27 \right) \end{aligned}$$

$$\begin{aligned} III\left( x \right)=2xI_{0}\left( x \right)+\left( -4+3x^{2} \right)I_{1}\left( x \right)+2xI_{2}\left( x \right)+x^{2}I_{3}\left( x \right)\#\left( 28 \right) \end{aligned}$$

$$\begin{aligned} YYY\left( x \right)=-2xY_{0}\left( x \right)+\left( 4+3x^{2} \right)Y_{1}\left( x \right)+2xY_{2}\left( x \right)-x^{2}Y_{3}\left( x \right)\#\left( 29 \right) \end{aligned}$$

$$\begin{aligned} KKK\left( x \right)=2xK_{0}\left( x \right)+\left( 4-3x^{2} \right)K_{1}\left( x \right)+2xK_{2}\left( x \right)-x^{2}K_{3}\left( x \right)\#\left( 30 \right) \end{aligned}$$

The expressions in the right-hand side matrix of (22) are the external force acting on the membrane, first of which is input voltage dependent and the second of which is caused by the incident sound pressure. Therefore, the constants we solve for in (22) are also related to both voltage (*V*) and acoustic pressure (*q*), so the final volumetric displacement (*W_vol_*) can also be directly divided into two terms related to voltage and acoustic pressure[^27^](#_ENREF_25):

$$\begin{aligned} W_{vol}=Y_{m}q+b_{t}V\#\left( 31 \right) \end{aligned}$$

where *Y_m_* is the volumetric displacement generated by per unit incident pressure; *b_t_* is the electromechanical transduction coefficient and is defined as the volumetric displacement by per unit input voltage.

$$\begin{aligned} Y_{m}=\frac{2\pi}{q}\left. \left[ \int_{0}^{a} W_{I}\left( r \right)rdr+\int_{a}^{b} W_{II}\left( r \right)rdr \right] \right|_{V=0} \#\left( 32 \right) \end{aligned}$$

$$\begin{aligned} b_{t}=\frac{2\pi}{V}\left. \left[ \int_{0}^{a} W_{I}\left( r \right)rdr+\int_{a}^{b} W_{II}\left( r \right)rdr \right] \right|_{q=0} \#\left( 33 \right) \end{aligned}$$

Therefore, the mechanical impedance and the electromechanical transduction ratio shown in Fig. S2 are derived as follows:

$$\begin{aligned} Z_{m}=\frac{1}{j\omega Y_{m}}\#\left( 34 \right) \end{aligned}$$

$$\begin{aligned} \eta=\frac{b_{t}}{Y_{m}}\#\left( 35 \right) \end{aligned}$$

The Equivalent circuit model established for the single circular pMUT is shown in Fig. S2, where *C_0_* is the blocked capacitance and it is calculated as follows^61^:

$$\begin{aligned} C_{0}=\frac{\varepsilon_{z}A_{e}}{h_{p}}\#\left( 36 \right) \end{aligned}$$

where *ε_z_* is the permittivity of piezoelectric material, *A_e_* is the area of top electrode and *h_p_* is the thickness of the piezoelectric layer. Then the electrical impedance can be derived as:

$$\begin{aligned} Z_{e}=\frac{1}{j\omega C_{0}}\#\left( 37 \right) \end{aligned}$$

And *Z_a_* is the acoustic radiation impedance which in the form as follows^40,41^:

$$\begin{aligned} Z_{a}=\frac{\rho c\left[ \left( 1-\frac{J_{1}\left( 2kb \right)}{kb} \right)+i\frac{H_{1}\left( 2kb \right)}{kb} \right]}{A}\#\left( 38 \right) \end{aligned}$$

where *k* is the wavenumber, *A* is the effective area of the diaphragm, *J_1_* is the first-order Bessel function of the first kind and *H_1_* is the first-order Struve function.

## Single cell characterization results

Both the EQC model and the finite element method (FEM) model calculated the volumetric displacement-frequency response in air and water, respectively and their results agree well with each other as shown in Fig. S3A. The resonant frequency in air is about 3.3 MHz, while in water it is about 2.7 MHz. And the calculation error of the EQC model is about 7%. To ensure that the device operates at the correct mode, we examined the mode at the resonant frequencies in water calculated by volumetric displacement-frequency response. As shown in Fig. S3B, pMUT operates in mode I at the frequency of 3.3 MHz in water. Then the effect of the coverage of the top electrode on the membrane vibration displacement was investigated. The electrode we chose is a circular inner electrode, and the displacement of pMUT vibration increases with the increase of electrode radius, and the vibration displacement of pMUT center reaches the maximum at the top electrode coverage ratio of 0.67. When the upper electrode size increases further towards covering the whole piezoelectric layer, the membrane displacement decreases to zero.


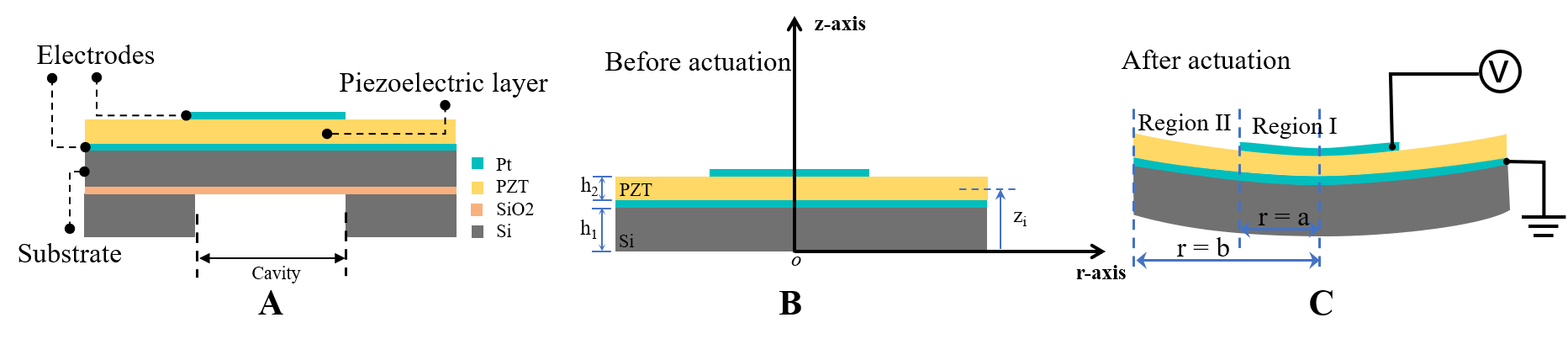


**Fig. S1** Scheme of the single piezoelectric micromachined ultrasound transducer (pMUT) cell. **A.** The SOI structure of the PZT pMUT. **B.** The pMUT cell before actuation and the adopted polar coordinate system. **C.** The piezoelectric layer actuated by an AC voltage and the pMUT divided by two regions. Region I is underneath the top electrode. Region II is the area beyond the top electrode.

**Table S1.** Piezoelectric transducers (conventional bulk PZT, 1-3 composite PZT, cMUTs, pMUTs) for in-air use, for monitoring or imaging the epidermis and deep tissues.

|  | Piezo. material | Freq. | Voltage | Application |
| --- | --- | --- | --- | --- |
| **Conventional**  **Transducer** | 1-3 composite PZT | 2MHz | 25 V | Continuous monitoring of deep-tissue hemo-dynamics**^8^** |
|  | 1-3 composite  piezoelectric material | 3,7,10 MHz | 40V | Long-term continuous imaging of diverse organs**^2^** |
|  | Bulk PZT | N/A | High-voltage | Epidermal patch：monitoring of hemo-dynamic and metabolic biomarkers**^36^** |
|  | PZT-5H | 2-6MHz | High-voltage | Monitoring deep tissues in moving subjects**^10^** |
|  | 1-3 composite PZT | 2.4MHz | 100 V | Photoacoustic patch：imaging of hemoglobin and core temperature**^37^** |
| **cMUT** | N/A(cMUT) | 0.443-4.16 MHz | 40 V bias | Portable applications in air**^34^** |
|  | N/A(cMUT) | 3.3-4.2 MHz | 7.4-25 V bias | N/A**^35^** |
| **pMUT** | PZT | 4.8 MHz | 20 V | 2D intracardiac echo (ICE)**^31^** |
|  | PMN-PZT | 5,10 MHz | 40V | Harmonic imaging and image-guided therapy**^38^** |
|  | (K, Na) NbO_3_ | 104.5 kHz | 2V | In-air haptic feedback, loudspeaker, and AR/VR systems**^33^** |
|  | PZT | 3 MHz | 5 V | Volumetric 3D imaging at 7cm depth (this work) |

**Table S2.**  Structural and material parameters of the single cell pMUT.

| Symbol | Value | Unit | Description |
| --- | --- | --- | --- |
| *a* | *b*$\times\alpha$ | μm | Top electrode radius |
| *b* | 200 | μm | Diaphragm radius |
| *h_1_* | 8 | μm | Si thickness |
| *h_2_* | 5 | μm | PZT thickness |
| *α* | 0.67 | / | Top electrode coverage ratio |
| *Y_1_* | 170 | GPa | Si Young’s modulus |
| *v_1_* | 0.28 | / | Si Poisson’s ratio |
| *ρ_1_* | 2329 | kg/m^3^ | Si density |
| *Y_2_* | 83.26 | GPa | PZT Young’s modulus |
| *v_2_* | 0.36 | / | PZT Poisson’s ration |
| *ρ_2_* | 7500 | kg/m^3^ | PZT density |
| *d_31_* | 123 | pm/V | PZT piezoelectric constant |
| *ε* | 950 | / | PZT Dielectric constant |


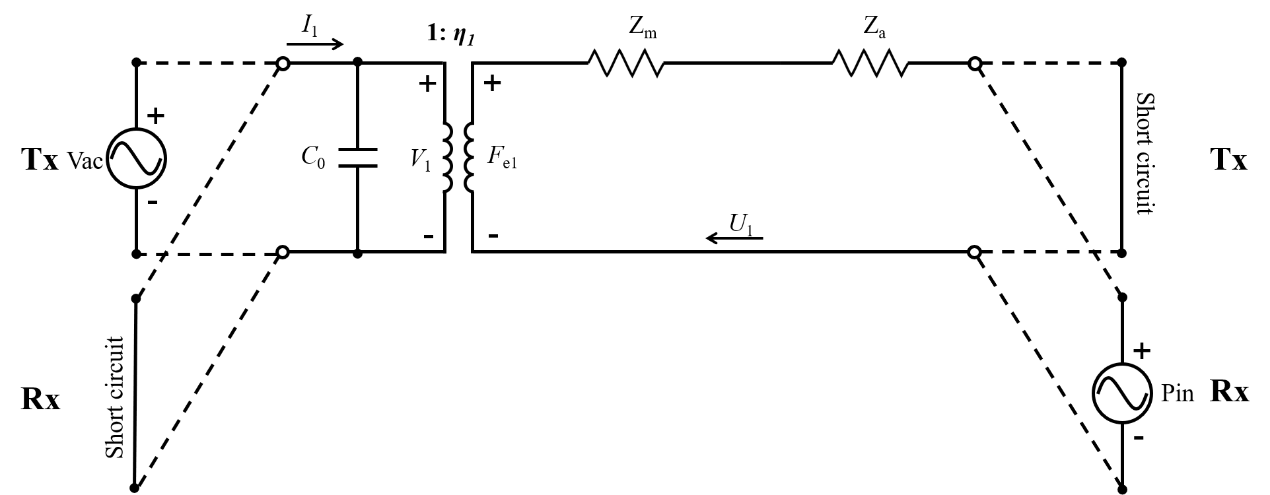


**Fig. S2.** Equivalent Circuit Model of the single piezoelectric micromachined ultrasound transducer (pMUT) cell.


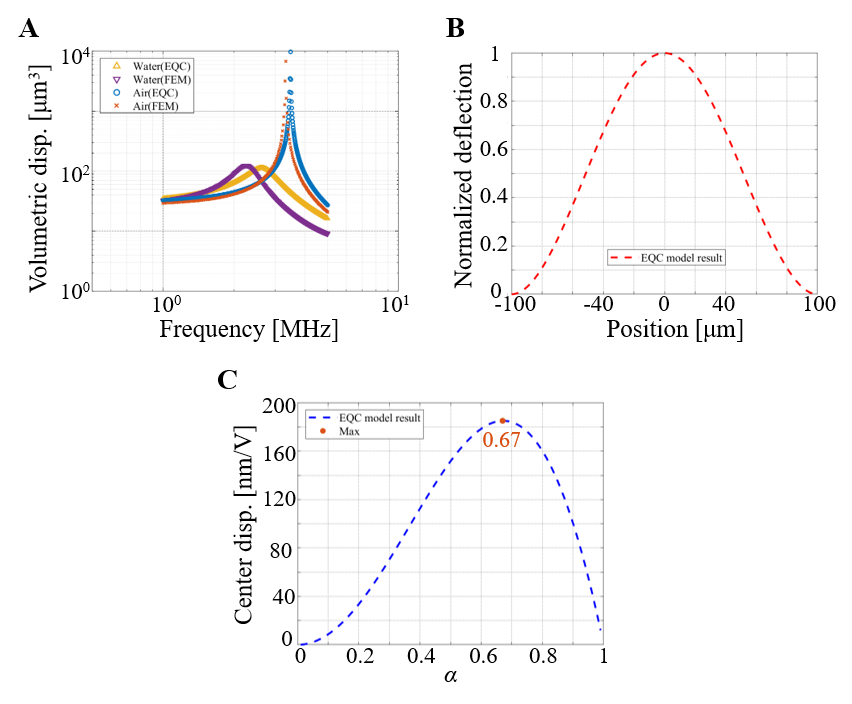


**Fig. S3.** **A.** Volumetric displacement-frequency response of the single cell PZT piezoelectric micromachined ultrasound transducer (pMUT) in air and water. **B.** The model shape at resonance of the single cell pMUT in water. **C.** Effect of top electrode coverage(α) on membrane center displacement.


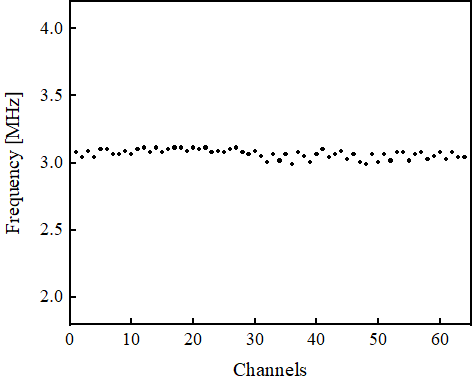


**Fig. S4.** Center frequency distribution characterization of the 8×8 piezoelectric micromachined ultrasound transducer (pMUT) phased array transducer with 64 elements (channels).


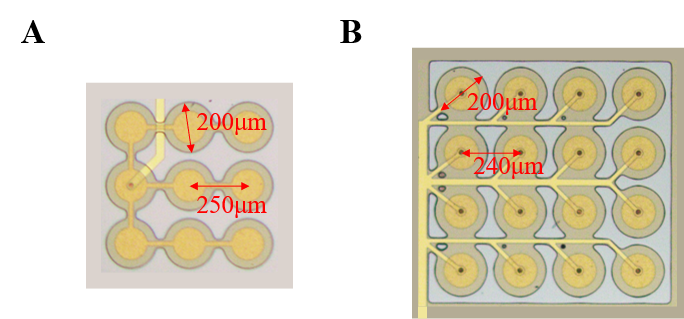


**Fig. S5.** Optical microscope photos of different layout elements. **A**. 3×3 layout. **B**. 4×4 layout.


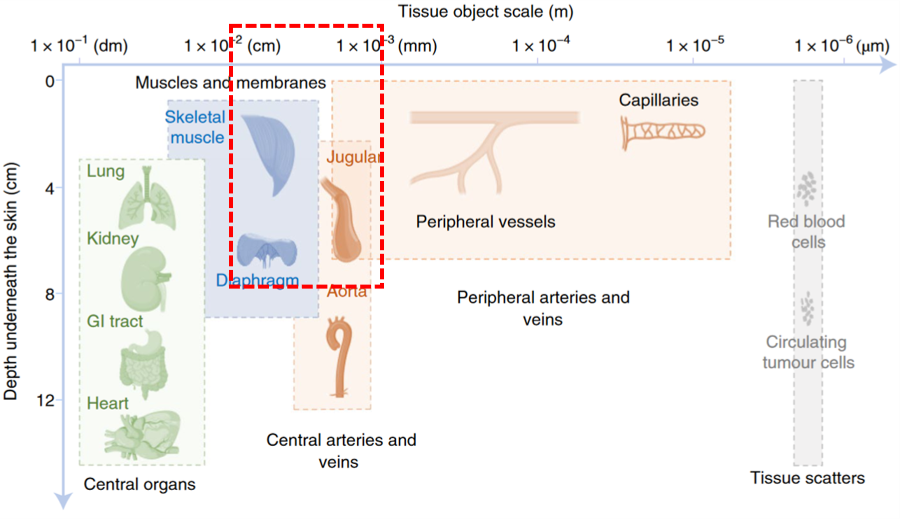


**Fig. S6.** Depths underneath the skin and dimensions of representative tissues and organs of interest[^8^](#_ENREF_8).
